# Estimating heterogeneous effects of internet use on environmental knowledge: Taking population heterogeneity into consideration

**DOI:** 10.1371/journal.pone.0288495

**Published:** 2023-07-12

**Authors:** Xiaoxiao Cheng

**Affiliations:** College of Media and International Culture, Zhejiang University, Hangzhou, Zhejiang, China; Beijing University of Technology, CHINA

## Abstract

The recent decades have witnessed the rise of digital media; as an essential informal way of environmental education, the internet has become an important source where public acquire environmental knowledge. The current study investigates the heterogeneous treatment effects of internet use on environmental knowledge across members of the Chinese population. Based on a nationwide survey in China, the propensity score approach, a series of statistical techniques that are often used in the counterfactual framework to understand the causal relationship between an intervention and an outcome, is employed to adjust for population heterogeneity and to estimate heterogeneous treatment effects. The findings reveal highly significant positive associations between internet access/use and environmental knowledge. More importantly, this study shows that individuals who are least likely to access the internet benefit most from the knowledge returns to internet access and use, indicating a positive outlook for the potential of the digital media to narrow the environmental knowledge gap.

## Introduction

Conventional wisdom has demonstrated the effectiveness of media in educating the public about the environment [1,2]. To a large extent, the media are important learning sources from which we can acquire environmental knowledge [3,4]. This is especially true in regard to the digital and social media landscape in recent decades [5], during which a wide range of environmental information has proliferated online [6].

The internet is not a single technology but a collection of technologies and communication infrastructure encompassing converged internet-based multipurpose capabilities and applications [7,8]. In this context, accessing environmental information through these multifunctional affordances has two-fold meanings, referring to both the adoption of the internet as an information system/platform and the adoption of diverse internet usage modalities [7]. Consequently, the internet serves as a conduit connecting environmental issues with a wider audience, granting people more opportunities to engage with and stay informed about the environment [4,9], which is vital for individuals across different social strata [10].

Despite numerous studies contributing valuable insights, the critical inquiry into the between internet access/use and environmental knowledge remains incomplete. Specifically, previous investigations of this area have failed to take into account the fact that there are digital divides [11,12] or access gaps [13] between people who are connected to the internet and those who are disconnected [14,15]. This omission is problematic as the likelihood of an individual accessing and using the internet is determined by a series of sociodemographic and sociocultural factors that are closely related to knowledge acquisition. Therefore, as suggested by Hu [16], if this pretreatment heterogeneity and the potential selection process it might trigger are not considered, the knowledge effects of the internet might be overestimated or underestimated. Additionally, since mechanisms influencing internet use may differ by social background, it is possible that knowledge gains vary by the probability of individuals’ access to the digital media. Such heterogeneous treatment effects, although they have been investigated and confirmed extensively in social inequality and/or discrimination studies [16–18], are largely unexplored by environmental communication scholars.

Against this backdrop, this study delves into the heterogeneous treatment effects of internet access/use on environmental knowledge among segments of the Chinese population. To this end, this study analyzes data from the 2013 Chinese General Social Survey (CGSS 2013) and employ the propensity score approach, which is a combination of a series of advanced analytical techniques for causal inference and the estimation of heterogeneous treatment effects with observational data. The results suggest that individuals who could benefit most (i.e., acquire more environmental knowledge) from the internet are those who are least likely to access and use it, indicating a positive outlook for the ability of the internet to narrow the environmental knowledge gap in China.

## Literature review

### Environmental learning and digital media

Existing literature on environmental learning has primarily concentrated on the implementation and effectiveness of environmental education as a means of informing the public about environment matters. Environmental learning, as suggested, is not exclusively institutionalized (e.g., environmental education organized by schools and government) but is socially learned through which a variety of media channels come into play [1,19,20].

Media plays a crucial role in mediating our understanding of environment issues. Compelling evidence indicates that both traditional and digital media have influenced the construction of a mediated reality surrounding the environment [6,21–23]. Environmental representation not only translate abstract and complex scientific findings about the environment into plain and easy-to-understand language [24], but also offer interpretations of scientific facts by incorporating and synthesizing information from various stakeholders, such as scientists and policymakers [2,25].

The swift proliferation of ICTs has resulted in a significant transformation in the media landscape and an increase in the adoption of internet- and social media-based interventions for environmental education [26,27]. Owing to their cost-free nature and relative efficacy in reaching target audiences, digital media, particularly the internet, have become increasingly integrated into our daily routines [28] and are viewed as the most cost-effective channels for environment communication and education [1,20,26,29,30]. Furthermore, the internet’s on-demand and crowd-sourcing features offer up-to-date environmental news, enable real-time interactions, and foster engagement and social participation [29,31], empowering audiences to exercise greater agency in environmental learning [20]. As highlighted by the concepts such as technology-enhanced learning and the “videophilia shift” [28,32], digital media technologies in general, and the internet in particular, are regarded as promising educational and communicative tools and are increasingly deployed in environmental education practices.

### Internet access/use and environmental knowledge

Extensive empirical research has investigated the impact of internet access and use on attitudinal [33–36] and behavioral [30,31,37,38] aspects of sustainability outcomes. A common finding from these studies is that environmental knowledge, as a cognition-based sustainability outcome, plays an important intermediary role between internet use/access and both environmental attitude and pro-environmental behavior [e.g., 30,37,38].

Nevertheless, the manner in which the internet shapes public knowledge about environment remains unclear and often overlooked [24]. Existing observations have not yielded a systematic or unified conclusion regarding the nexus between internet use/access and environmental knowledge. While numerous studies have illustrated the strong positive effects of internet-enabled free-choice and experiential learning [39] on overcoming the limited cognition barrier to sustainability and acquiring environmental knowledge [e.g., 8,31,37,40], significant disparities persist in the magnitude and direction of these effects. Taddicken [24], for example, reported a non-significant relationship between everyday internet use and climate change knowledge, a pattern that also applies to climate change-specific internet use. Moreover, daily internet use was found to adversely impact the levels of climate change knowledge among users critical of media reporting on climate change [24].

The mixed results mentioned above can be partially attributed to the oversimplification of employed conceptual frameworks, which only capture superficial effects of internet use/access without explaining the underlying mechanisms [37]. In other words, the internet use-environmental knowledge association is not necessarily self-evident or straightforward but can be nuanced and indirect. As Taddicken [24] pointed out, the internet may promote environmental knowledge “not in the sense of being a tool for creating a more informed citizenry…but in the sense of playing a crucial role in activating and affecting the perception” of environmental issues.

Researchers have increasingly focused on developing novel conceptual models to uncover potential pathways through which internet use influences sustainability outcomes [e.g., 37,38]. Central to these models is the consideration of the intricate relationship between online media content and audience reception [41]. Negativity bias theory [42,43] has been applied to identify cognitive and perceptual factors that serve as intermediaries between internet use and sustainability outcomes, with perceived environmental threats (PET), satisfaction with governmental environmental protection (SGEP), and environmental awareness (EA) among the most notable mediators. The present study, therefore, incorporates these three factors to elucidate the intricate mechanisms linking internet use to environmental knowledge.

Negativity bias theory posits that people tend to assign greater significance to negative news in comparison to positive and neutral information [44,45]. This predisposition could lead individuals to pay more attention to negative information about environmental degradation and pollution [46], making people more conscious of the environmental issues and enhancing their environmental awareness [34,38]. Substantial evidence supports a significant and positive relationship between internet use and environmental awareness [30,31,33,47]. Scholars suggest that this heightened awareness subsequently contributes to enhanced environmental knowledge [24,39,48,49]. In short, environmental awareness, as a cognitive factor, may function as a mediator in the association between internet use and environmental knowledge. Therefore, the research hypothesis is as follows:

**H1**: Environmental awareness plays a mediating role between internet use and environmental knowledge.

Perceived environmental threats and satisfaction with governmental environmental protection are also considered parallel perceptual mediators in the impact of internet use on environmental knowledge. The former refers to an individual’s subjective perception of environmental risks or threats [38], while the latter represents an individual’s evaluation of satisfaction with government actions and policies aimed at environment protection [42]. Scholars [37] highlight two types of negative prejudice that emerge when individuals access and consume online negative environmental information: internet use not only intensifies and exacerbates individuals’ judgments on the severity of environmental problems but also erodes their trust in the government and satisfaction with governmental environmental protection efforts. Convincing evidence demonstrates the promoting effect of internet use on perceived environmental threats [38,46,50] and the inhibiting effect on resident’s satisfaction with governmental environmental protection [37,42]. Moreover, sporadic evidence reveals significant relationships between environmental knowledge and these two perceptual factors [e.g., 37,51]. The analysis above suggests that perceived environmental threats and satisfaction with governmental environmental protection may act as intermediaries in the relationship between internet use and environmental knowledge. Drawing on the reviewed literature, this study proposes the following hypotheses:

**H2**: Perceived environmental threats play a mediating role between internet use and environmental knowledge.
**H3**: Satisfaction with governmental environmental protection plays a mediating role between internet use and environmental knowledge.

### The heterogeneous effects of the internet use/access on environmental knowledge

As previously mentioned, the observed inconsistency in the relationship between internet use and environmental knowledge may be attributed to the oversimplified specification of their association. Another key cause, from a methodological standpoint, is the utilization of mean-based homogeneous analytical methods [52], which fail to capture the full scope of the heterogeneous effects of internet use and access.

Variability across units of analysis is inherent in all social phenomena, which also applies to the investigation of digital media effects [53] on sustainability outcomes. In recent years, an emerging body of research has focused on exploring the heterogeneous effects of internet use/access on pro-environmental behavior [30,31]. Furthermore, scholars have employed quantile regression models to examine the influence of ICTs adoption on reducing agrochemical inputs [52] and enhancing economic and production performance in sustainable agriculture practices [54] across various quantiles. Despite these pioneering studies have tapped into certain aspects of the heterogenous effects of the internet, this research area remains nascent, necessitating further and methodologically rigorous investigations to achieve a comprehensive and holistic understanding of the phenomenon.

The methodological literature and evaluation research apply the label “heterogeneous treatment effect” or “treatment effect heterogeneity” to describe situations where the impact of a treatment varies across study subjects [55–57]. This variability can be ascribed to pretreatment heterogeneity.

Pretreatment heterogeneity refers to observed and/or unobserved individual-level attributes that affect a subject’s treatment status (treated or untreated) and the outcome of interest [55,56]. Selection bias arise when systematic differences in pretreatment heterogeneity occur across units [58,59]. Given the self-selective nature of the internet [24], individuals’ decision to be internet users or non-users are based on an array of baseline characteristics [52,54] that also influence treatment outcomes. Consequently, observed heterogeneous effects of internet access and use may be confounded by differences in these antecedents and the resulting selection bias. In other words, it is the combination of internet access and the differential propensity to access it—summarized as a function of these baseline characteristics—jointly affect treatment outcomes. Prior studies have demonstrated that sociodemographic factors such as age, education, gender, race, income, and profession [14,60], as well as sociocultural factors like perceived characteristics of the internet (PCI) and perceived popularity of the internet (PPI) [61,62], significantly correlated with internet access and use.

It is noteworthy that pretreatment heterogeneity can also affect the relationship between treatment and its outcomes, potentially leading to differential susceptibility to an intervention among various social segments and thus contributing to heterogeneous treatment effects. This is exemplified by the prevalence of digital media-driven environmental knowledge gaps in general [1,63] and in specific environmental domains, such as biodiversity [51], water [64], climate change [65], and natural disasters [66]. The original knowledge gap theory [67] highlights the uneven distribution of knowledge gains among different social segments. According to this theory, the observed systematic-level social differential in knowledge stem from the varying impacts of internet use between population segments, which result from individual-level differences in educational attainment, a proxy measure of socioeconomic statues (SES) [19,68,69]. The rationale for SES-induced knowledge gaps is evident, partly because highly educated people possess greater prior knowledge and stronger cognitive abilities, facilitating information processing and recall, and enabling them to acquire more knowledge than their less-educated counterparts [19,68]. Disparities in knowledge accumulation and information acquisition have also been observed along various sociodemographic lines, such as, age, race, ethnicity, and gender [70]. Additionally, income has been identified as a factor contributing to gaps in environmental information acquisition among different groups [31]. As observed, low-income individuals have limited ways to acquire information, making them more sensitive and susceptible to online environmental information compared to middle- and high-income individuals.

In summary, pretreatment heterogeneity, the potential selection bias it may induce (i.e., the propensity to access the internet due to differences in antecedents), and the treatment conditions (internet access or lack thereof) collectively contribute to heterogeneous effects of internet access on environmental knowledge among subpopulations. Consequently, it is pivotal and nontrivial for the present study to employ a nuanced analytical approach capable of capturing the net effect of internet access and use on environmental knowledge, thereby advancing a more comprehensive understanding of the relationship under investigation.

Therefore, this study endeavors to address the following three research questions (RQs):

**RQ1**: How does pretreatment heterogeneity affect the probability of internet access? Are there any patterns in internet access by sociodemographic and sociocultural factors?
**RQ2**: Does internet access exert a positive, negative, or neutral influence on environmental knowledge?
**RQ3**: Are environmental knowledge gains distributed differentially among various population segments with distinct baseline characteristics and varying probabilities of accessing the internet? If so, what is the pattern of this distribution?

## Methods

### Estimation and analytical strategy

The propensity score matching (PSM) method [17,55–57,71] is employed to address the selection bias originating from the endogeneity issue of internet access variable [54], facilitating accurate estimation the heterogeneous treatment effects of internet access and use on environmental knowledge. The fundamental principle of PSM revolves around mitigating or even removing bias due to pretreatment heterogeneity and balancing the observed baseline characteristics of the treatment and control groups, rendering them comparable. Within PSM, the propensity score denotes the conditional probability of receiving the treatment, as expressed by the following equation:

p(X)=Pr[D=1|X]=E[D|X], (1)

where *X* represents a vector of observed covariates, and *D* is a treatment dummy. In this investigation, the propensity score *p*(*X*) corresponds to the respondent’s likelihood of accessing the internet (*D* = 1), encapsulating all relevant information connecting the array of baseline characteristics, including the sociodemographic (e.g., age, education, and income) and sociocultural (e.g., PPI and PCI) factors, which jointly determine the probability of internet access and the subsequent environmental knowledge acquisition.

Based on the counterfactual framework [72–74] and estimated propensity scores, PSM identifies a counterfactual control group similar to the treatment group via various matching algorithms [30,75]. In this study, the 4-nearest neighbor matching method is used to generate pairs of treatment-controlled samples sharing similar characteristics. This procedure emulates a quasi-experimental setting [16,18,72], allowing for the estimation of the net effect of internet access on environmental knowledge as follows:

$$\begin{array}{l} ATT=E(Y_{i}^{1}-Y_{i}^{0}|D_{i}=1)\\ \;=E\{E(Y_{i}^{1}-Y_{i}^{0}|D_{i}=1,p(X_{i}))\}\\ \;=E\{E[Y_{i}^{1}|D_{i}=1,p(X_{i})]-E[Y_{i}^{0}|D_{i}=0,p(X_{i})]|D_{i}=1\}, \end{array}$$ (2)

where the treatment effect, i.e., the average treatment effect on the treated group (ATT), represents the average difference in treatment status among the individuals who are actually treated, with $Y_{i}^{1}$ and $Y_{i}^{0}$ denoting the potential outcomes of the treatment and control groups, respectively.

In addition to PSM, this study incorporates the parallel multiple mediation model [76] and utilizes bootstrapping techniques (PROCESS macro for SPSS, Version 4.3) to test the plausible mediating effects of environmental awareness (H1), perceived environmental threats (H2), and satisfaction with governmental environmental protection (H3).

### Data and sampling

The CGSS is a nationwide, repeated, cross-sectional social survey; the data we use came from the 2013 wave, which covered all mainland provinces and municipalities and used a multistage probability proportional to size (PPS) stratified sampling design. The CGSS 2013 sampled 11,483 individuals with a response rate of 72.17% (in-person interviews). We selected our cases from the central (age, education, gender, occupation, etc.), lifestyle (internet access), and environment (environmental knowledge) modules. Ultimately, we generated a valid original/unmatched sample of 9,544 cases for the purposes of this study. Approximately 50.11% of the respondents were male, and the average age of the whole sample was 43.63.

### Measurement

Environmental knowledge. The CGSS 2013 included a 10-item dichotomous Environmental Knowledge Scale (items B25), which has shown adequate measurement quality in previous studies. Respondents were asked, for example, whether they thought the statement “acid rain has nothing to do with burning coal” was right or wrong. Correct answers were coded as 1, while incorrect and “don’t know” answers were coded as 0. I added up all 10 items to construct an aggregated environmental knowledge score ranging from 0 to 10 (*M* = 4.94, *SD* = 2.82); the statistics show that this scale has good measurement reliability (*KR-20* = .817). The full list of questions and descriptive statistics of each item are presented (see S1 Fig).

Internet access. Respondents were asked how often they used the internet (including mobile internet use) in the past year on a 5-point scale ranging from 1 (never use) to 5 (always use). To measure internet access, the treatment variable of this study, I recoded the responses as “0 = No access” and “1 = Access”. The data show that 50.54% of the respondents accessed the internet.

Mediating variables. Environmental awareness (EA) is determined by respondents’ opinions on the importance of environmental issues among the top three social problems (out of 10). A 4-point Likert scale (1 = not chosen, 4 = top social problem) measures the degree of EA. Perceived environmental threats (PET) were measured by asking respondents to report their perceptions of the severity of twelve environmental issues (e.g., air, water, and noise pollution) in their living area on a scale from 1 (not a problem) to 5 (extremely serious). The PET score was calculated by averaging these item scores. Satisfaction with governmental environmental protection (SGEP) was measured by asking respondents to evaluate the efforts of both central and local governments in protecting the ecological environment and combating pollution on a scale ranging from 1 (terrible) to 5 (excellent). The SGEP score was calculated by averaging the scores of these two items.

Pretreatment covariates. The baseline characteristics used to predict the propensity scores are labelled as “pretreatment covariates” to differentiate them from the control variables when analyzing the effects of internet use on environmental knowledge. In line with prior studies, I selected a list of sociodemographic and sociocultural factors, including gender, age, education, residence, occupation, family income, marital status, innovativeness, and social contact to estimate individuals’ propensity scores. All these measures have been found to concurrently affect internet access as previously mentioned, and most of the baseline characteristics have also been shown to be directly correlated with environmental knowledge in environmental sociology studies [77–79]. The measures of gender, residence, occupation, and marital status were straightforward. Family income was measured as the average annual household income in 2012 (in Chinese Yuan) and was natural-log transformed to correct for its positive skewness. Age was categorized into five groups (i.e., 18–25, 26–35, 36–45, 46–55, and 56–65). Education was converted into years of schooling (0 = not educated; 6 = primary school or private school; 9 = junior middle school; 12 = high school, including secondary school, technical school, and vocational high school; 15 = junior college; 16 = undergraduate; and 19 = master’s degree or above) and was divided into four groups (i.e., less than primary school, middle school, high school, and college and above). Due to secondary data limitations, individuals’ innovativeness was employed as a proxy measure for PCI. Innovativeness was assessed by asking the number of traditional Chinese festivals respondents celebrated; the number they chose plus 1 represented the degree of innovation (ranging from 1 to 10). A respondent was considered innovative if the number equaled 1. Furthermore, I measured social contact as a proxy variable of PPI. Based on two items asking about respondents’ frequency of interaction with their friends and neighbors (from 1 = never to 7 = almost every day), I conducted an exploratory factor analysis to obtain the factor score as the social contact score.

Control variables. I selected several control variables, including generalized trust (1 = completely distrust to 5 = completely trust), social well-being (1 = very unwell to 5 = very well), sense of fairness (1 = grossly unfair to 5 = fairly fair), Chinese Communist Party (CCP) membership, ethnic identity, traditional media use (1 = never use to 5 = always use), and attention to environmental information via traditional media (1 = never to 3 = constantly).

## Results

### Descriptive statistics

Descriptive results for the unmatched sample (*N* = 9,544), categorized by treatment status (internet access or no internet access), are presented in Table 1. The findings reveal an extremely unbalanced sample between respondents who access or not access to the internet. Specifically, younger educated men who live in urban areas and have relatively higher occupational prestige are more likely to connect to the Internet. However, rural married women who have children, are old and are less educated are more likely to be excluded from cyberspace. Notably, internet adopters (*M* = 6.13, *SD* = 2.52) are more knowledgeable about the environment compared with individuals who do not access the internet (*M* = 3.71, *SD* = 2.58). The results of the descriptive analysis provide preliminary support for the impacts of pretreatment heterogeneity.

**Table 1:** Descriptive statistics by internet access based on unmatched sample.

Variables	No internet access (*N* = 4,720)		Internet access (*N* = 4,824)	
	Mean (% of total)	Std	Mean (% of total)	Std
***Dependent variable***				
Environmental knowledge	3.71	2.58	6.13	2.52
***Pretreatment covariates***				
Gender	0.53	0.49	0.47	0.49
Male (0)	(23.27)		(26.84)	
Female (1)	(26.18)		(23.70)	
Age	50.81	9.69	36.61	11.69
18–25	(0.41)		(10.34)	
26–35	(3.19)		(15.09)	
36–45	(10.79)		(13.45)	
46–55	(15.76)		(7.55)	
56–65	(19.31)		(4.11)	
Educational level (year)	6.97	3.72	11.93	3.32
Less than primary school	(25.32)		(3.57)	
Middle school	(17.34)		(14.28)	
High school	(5.84)		(15.35)	
College and above	(0.95)		(17.34)	
Residence type	0.28	0.45	0.59	0.49
Agricultural (0)	(35.49)		(20.28)	
Non-Agricultural (1)	(13.97)		(30.26)	
Occupation				
Never worked	(1.47)		(4.80)	
Farmer	(19.74)		(2.79)	
Self-employed	(11.53)		(11.59)	
Enterprise staff	(12.56)		(22.58)	
Government official	(3.68)		(8.54)	
Others	(0.48)		(0.25)	
Average annual household income (Ln)	9.22	0.97	9.91	0.87
Marital status	0.98	0.15	0.77	0.42
Unmarried (0)	(1.20)		(11.76)	
Married (1)	(48.25)		(38.79)	
Social contact	0.01	1.05	0.03	0.89
Innovativeness	4.04	1.96	3.96	1.96
***Control variables***				
Generalized trust	3.32	1.03	3.18	1.02
Happiness	3.67	0.88	3.81	0.77
Sense of fairness	3.04	1.05	2.85	1.01
Chinese Communist Party (CCP)	0.05	0.23	0.13	0.34
Non CCP member (0)	(46.79)		(43.98)	
CCP member (1)	(2.66)		(6.57)	
Ethnic identity	0.11	0.31	0.07	0.25
Han (0)	(44.16)		(47.13)	
Minorities (1)	(5.29)		(3.42)	
Traditional media use (radio, television, and newspaper)	-0.18	0.49	0.18	0.54

### Pretreatment heterogeneity and internet access

**Fig 1:**
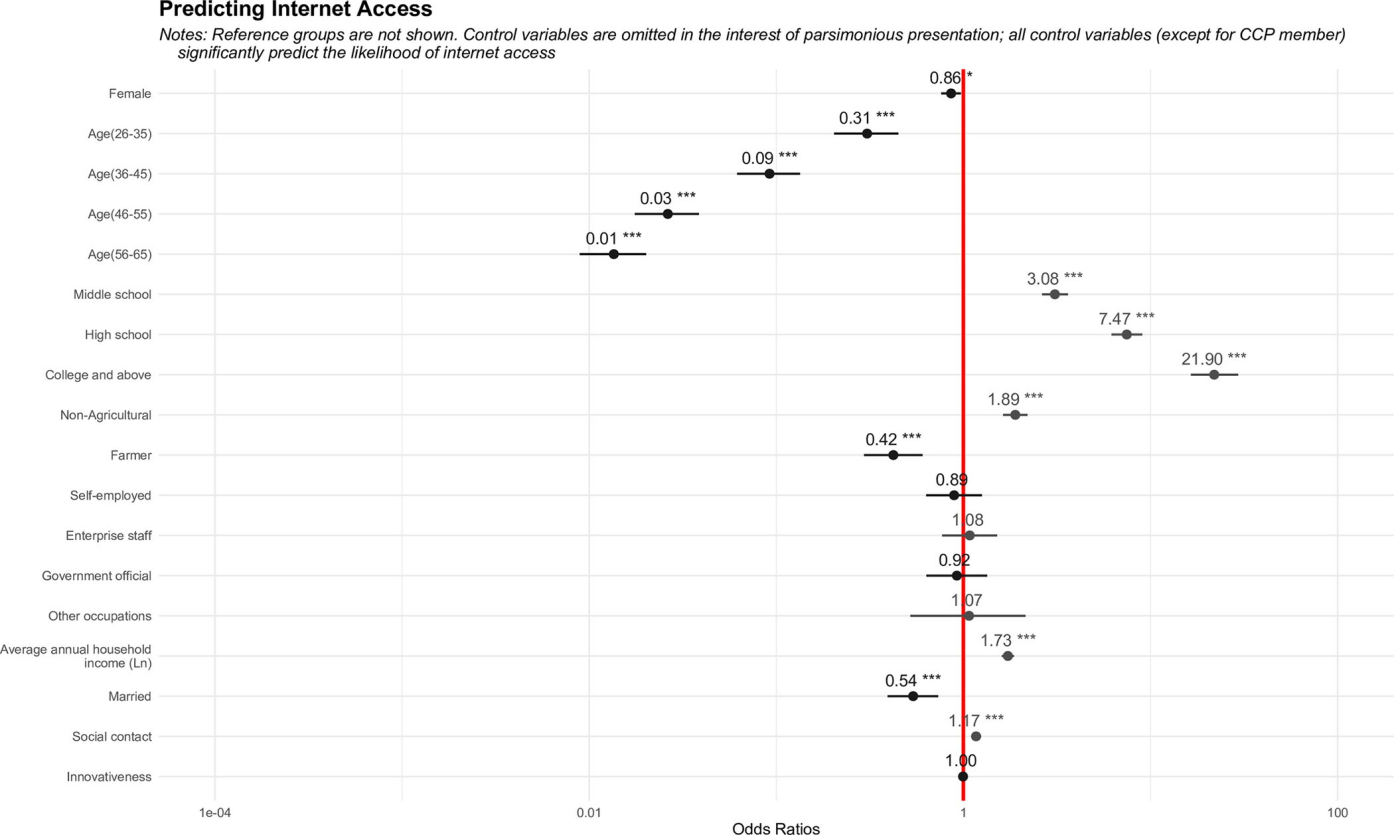
Logistics models predicting internet access for generating estimated propensity scores.

RQ1 asked how pretreatment heterogeneity affects an individual’s choice of internet access. To answer this question, a binary logistic model predicting internet access was conducted; the results further support the preliminary judgments derived from descriptive statistics. As shown in Fig 1, the likelihood of accessing the internet is conditioned on the observed baseline characteristics that being selected (except for innovativeness). The odds of women accessing the internet are only approximately 0.86 times the odds of men accessing the internet, ceteris paribus. Furthermore, compared to individuals who are older and less educated, have less social contact, live in rural areas as farmers and are not well off, the other individuals have greater propensity of accessing the internet.

### Effects of internet access on environmental knowledge

RQ2 questioned the environmental knowledge benefits from internet access among the Chinese population. The mean-based OLS regression model is performed. The findings based on the unmatched sample are presented in Model 1 (see Table 2). As shown in Model 1(a), the result indicates that internet access is significantly and positively correlated with environmental knowledge (*β* = 0.739, *p* <0.001); as shown in Model 1(b), such an effect is still prominent (*β* = 0.574, *p* <0.001) after all the pretreatment and control variables are adjusted for.

**Table 2:** OLS estimates of effects of internet access on environmental knowledge.

Variables	Model 1 (unmatched, *N* = 9,544)		Model 2 (matched, *N* = 9,379)	
	(a)	(b)	(a)	(b)
Internet access	0.739*** (0.070)	0.574*** (0.069)	0.735*** (0.070)	0.570*** (0.069)
Pretreatment and control variables	NO	YES	NO	YES
Intercept	2.447*** (0.279)	1.704*** (0.314)	2.530*** (0.286)	1.750*** (0.319)
*R* ^*2*^	0.3135	0.3438	0.3055	0.3367

Notes

Numbers in parentheses are robust standard errors.

*p < 0.05; **p < 0.001; ***p < 0.001 (two-tailed tests).

It is noteworthy that the effects derived from the unmatched sample represent the effect of internet access without considering the potential selection bias induced by pretreatment heterogeneities. In order to examine the existence and extent of potential selection bias, I perform the same analysis using the matched sample and compare its results with those derived from the unmatched.

**Fig 2:**
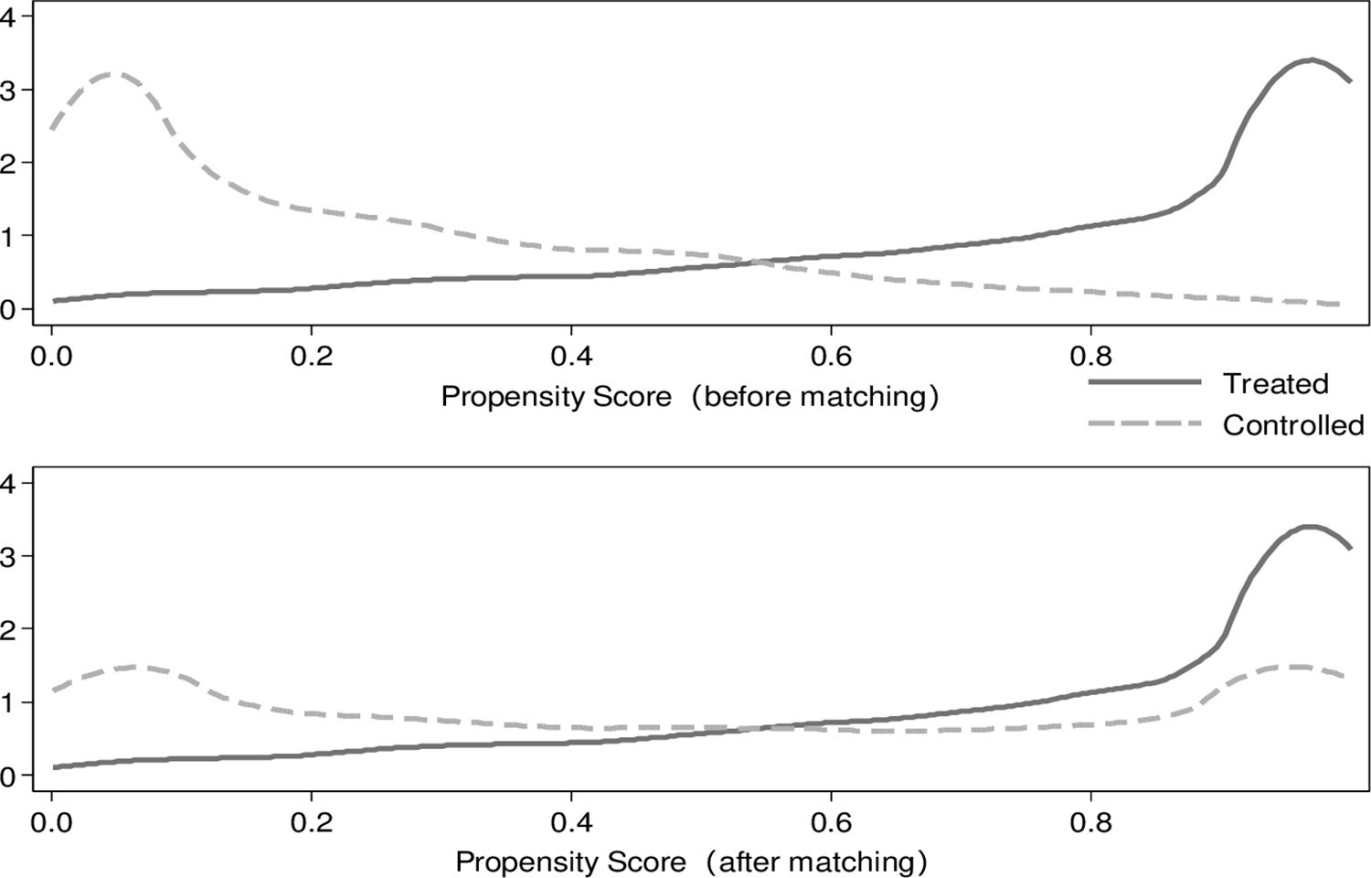
Kernel density (before and after PSM) of the treatment and control groups.

The matched sample is constructed by using the PSM method. Based on the logistic model that predicts choice of internet access, the individual-level propensity score, i.e., the probability of accessing the internet, for each respondent is predicted; these estimated propensity scores are used to balance the distribution of covariates between segments who had internet access (i.e., the treatment group) and those who did not (i.e., the control group). Next, I conduct PSM using a 4-nearest neighbor matching algorithm. A total of 165 cases are excluded because they are not in the “common support”; that is, they cannot match their potential counterparts in the treatment or control group due to their extreme propensity scores. Finally, a matched sample of 9,379 cases is obtained. The kernel density functions of the two groups are much closer after matching (as shown in Fig 2), indicating that the baseline characteristics in the two groups are similar.

The effect of internet access considering pretreatment heterogeneity and the resulting selection bias is summarized in Model 2 (see Table 2). The result indicates that the coefficient in Model 2(a) is smaller than that in Model 1(a), indicating a 0.4% inflation of the effect of internet access on environmental knowledge for the unmatched sample. This inflation suggests that the effect is slightly overestimated when pretreatment heterogeneity and selection bias are not accounted for; more importantly, it suggests the importance of balancing pretreatment covariates using PSM.

### Heterogeneity analysis

RQ3 deals with the question of whether knowledge gains from internet access vary among different groups with differential odds of accessing the internet and different baseline characteristics. Table 3 presents three treatment estimates based on PSM, including average treatment effect on the treated group (ATT, i.e., the net effects of internet access), average treatment effects (ATE) among the whole population, and the average treatment effect on the untreated (ATU, the average difference by treatment status among the members who are not treated). The results show that ATT < ATE < ATU, indicating heterogeneous treatment effects of internet access on environmental knowledge. More importantly, the results seem to suggest a negative distribution of environmental knowledge returns to internet access: the effects of internet access on environmental knowledge are the weakest among the treatment group whereas the untreated (i.e., respondents who have no internet access) benefit most from the internet if they gain access to and use it.

**Table 3:** PSM estimates of heterogeneous treatment effects of internet access on environmental knowledge.

Treatment Effect	Mean		Difference (coefficient)	Bootstrap S.E.
	Treated	Controlled		
ATT	6.081	5.391	0.689**	0.280
ATU	3.719	4.532	0.814***	0.135
ATE			0.752***	0.155

Notes

Standard errors are calculated using bootstrap with 500 replications.

*p < 0.05

**p < 0.001

***p < 0.001 (two-tailed tests).

### Mediating effect analysis

**Fig 3:**
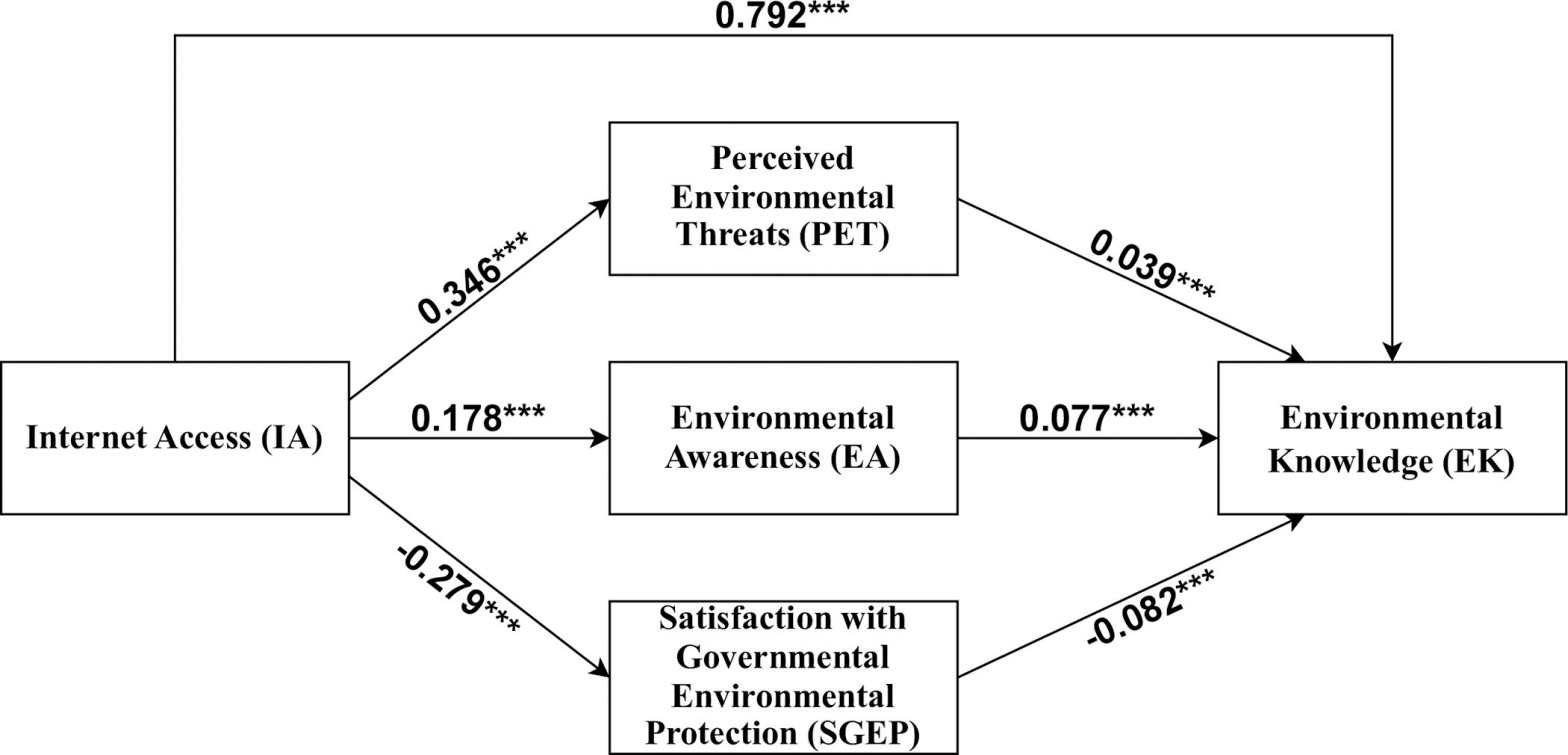
Multiple parallel mediation model and mediating effect results. Notes: ***p < 0.001. Path coefficients are standardized.

As shown in Fig 3, IA is positively associated with PET (*β* = 0.346, *p* < 0.001) and EA (*β* = 0.178, *p* < 0.001), both of which have a significant positive influence on EK (*β* = 0.039 and 0.077, *p* < 0.001). However, IA was found negatively related to SGEP (*β* = -0.279, *p* < 0.001), which further adversely impacts on EK (*β* = -0.082, *p* < 0.001). Bootstrapping test confirmed positive and significant (i.e., the 95% confidence interval does not contain zero) mediating effects for PET, EA, and SGEP. These findings indicate that perceived environmental threats, environmental awareness, and satisfaction with governmental environmental protection do partially mediate the effect of internet access on environmental knowledge. H1, H2, and H3 are therefore supported.

## Robustness checks

Given far too limited attention has been paid to the heterogeneous effects of the internet access/use on sustainability, the results need to be interpreted with caution. Two sets of additional robustness checks are performed.

### Addressing the bias due to sample selection in PSM

PSM relies on the assumption of common support, which plays a crucial role in ensuring that each treated unit can be matched with a comparable control unit. When the propensity scores for treated and untreated do not overlap, the unplaced groups are excluded, thereby potentially leading to sample selection bias [80]. As previously mentioned, a total of 165 unplaced observations, including 9 untreated and 156 treated, are dropped due to their extreme propensity scores. Since different PSM algorithms and procedures tend to produce a subsample that differ from the original population, this study examines the extent to which sample selection bias could influence the external validity of PSM, specifically, the generalizability of the results to the original sample using the matched data. Table 4 presents the PSM estimates based on two other types of matching algorithms are less sensitive to the lack of common support: kernel matching and local linear regression matching. The results also indicate a clear pattern of ATT < ATE < ATU, which is consistent with the estimates presented in Table 3, suggesting that the result is robust.

**Table 4:** PSM estimates based on kernel and local linear regression matching methods.

Matching method	Treatment Effect	Mean		Difference (coefficient)
		Treated	Controlled	
Kernel matching	ATT	6.080	5.259	0.822***
	ATU	3.719	4.578	0.859***
	ATE			0.840***
Local linear regression matching	ATT	6.081	5.417	0.664**
	ATU	3.719	4.519	0.800***
	ATE			0.732***

Notes

Standard errors are calculated using bootstrap with 500 replications.

*p < 0.05

**p < 0.001

***p < 0.001 (two-tailed tests).

In addition, stratification or subclassification could be used to alleviate the sample selection bias caused by the lack of common support, since its main focus is to compare treated and control units within each stratum. As suggested by previous research [57], the Stratification-Multilevel (SM) method is employed to verify the results. First, I group respondents into 15 propensity score strata such that people within the same stratum have close propensity score values (i.e., a similar likelihood of using the internet) and similar pretreatment conditions (i.e., the pretreatment covariates do not significantly differ between the treated and untreated groups within each stratum, see S1 Table). Notably, I order these strata according to the stratum-wise propensity scores to ensure that the likelihood of accessing the internet monotonously increases across strata [72]. Second, I estimate propensity score stratum-specific treatment effects within strata and obtain level-1 estimates. Third, I use an HLM model to regress the level-1 estimates of each stratum and obtain the level-2 slope across all the strata rank, which indicates the pattern of heterogeneous internet use/access effects on environmental knowledge.

**Fig 4:**
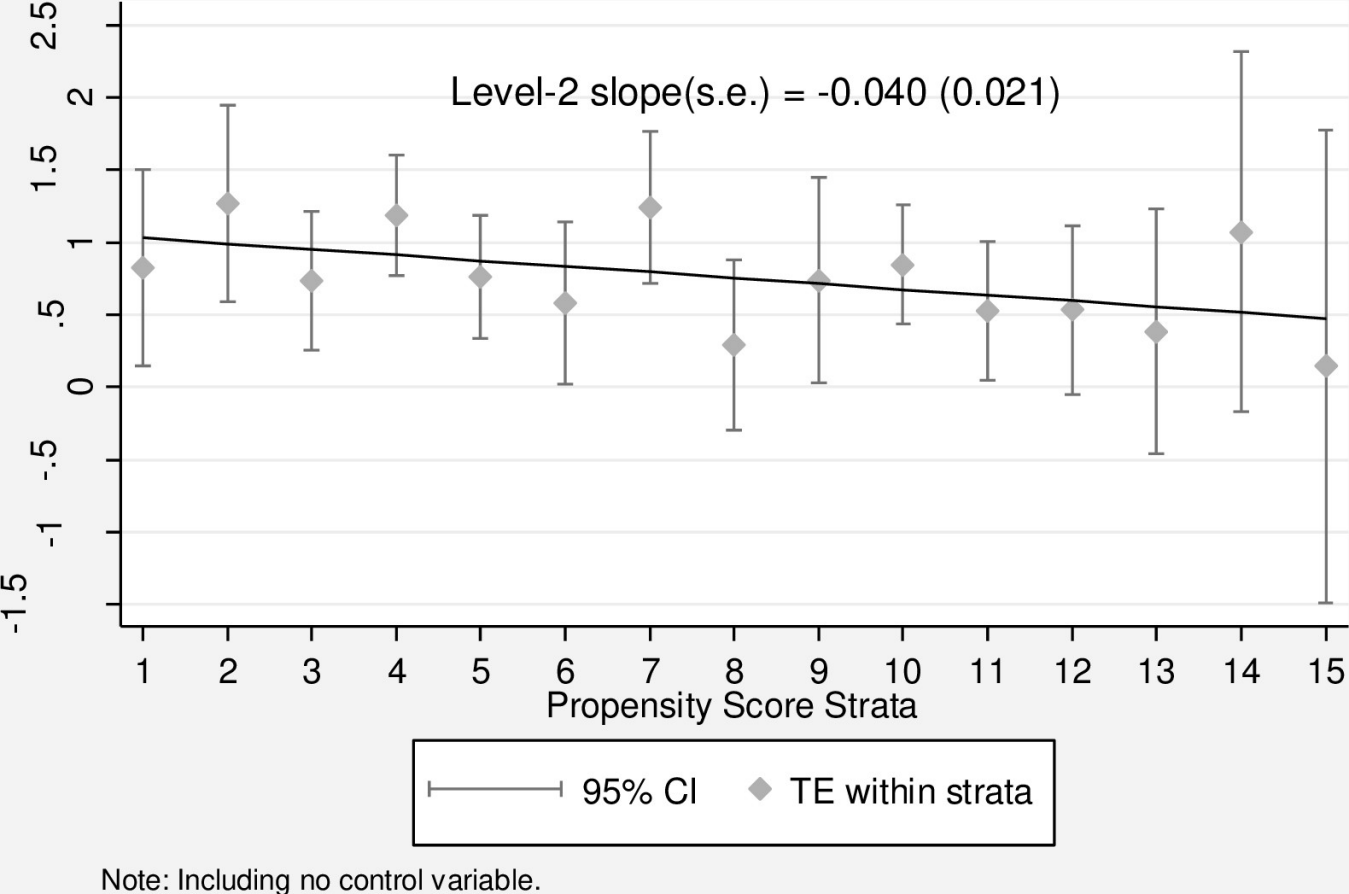
Heterogeneous treatment effects of internet access on environmental knowledge (SM method).

Once the sample is balanced, we can examine heterogeneous treatment effects as a function of the propensity score. Fig 4 depicts the pattern of heterogeneous treatment effects of internet access. The points in this figure represent the estimates of the stratum-specific effects of internet access on environmental knowledge. The significant downward linear slope illustrates a negative distribution pattern of environmental knowledge returns, suggesting that the knowledge returns to internet access decrease as individual’s propensity of internet access increase and that when individuals with a low probability of accessing the internet connect to the internet, they may benefit the most from doing so. The Matching-Smoothing (MS) method is also employed to for further verification; the result derived (see S2 Fig) has the similar pattern as that in Fig 4. In summary, this negative pattern sheds light on the heterogeneous effects of internet access on environmental knowledge, as well as the potential of internet to close the environmental knowledge gaps.

### Considering the problem of unobserved confounders and other endogeneity issue

**Fig 5:**
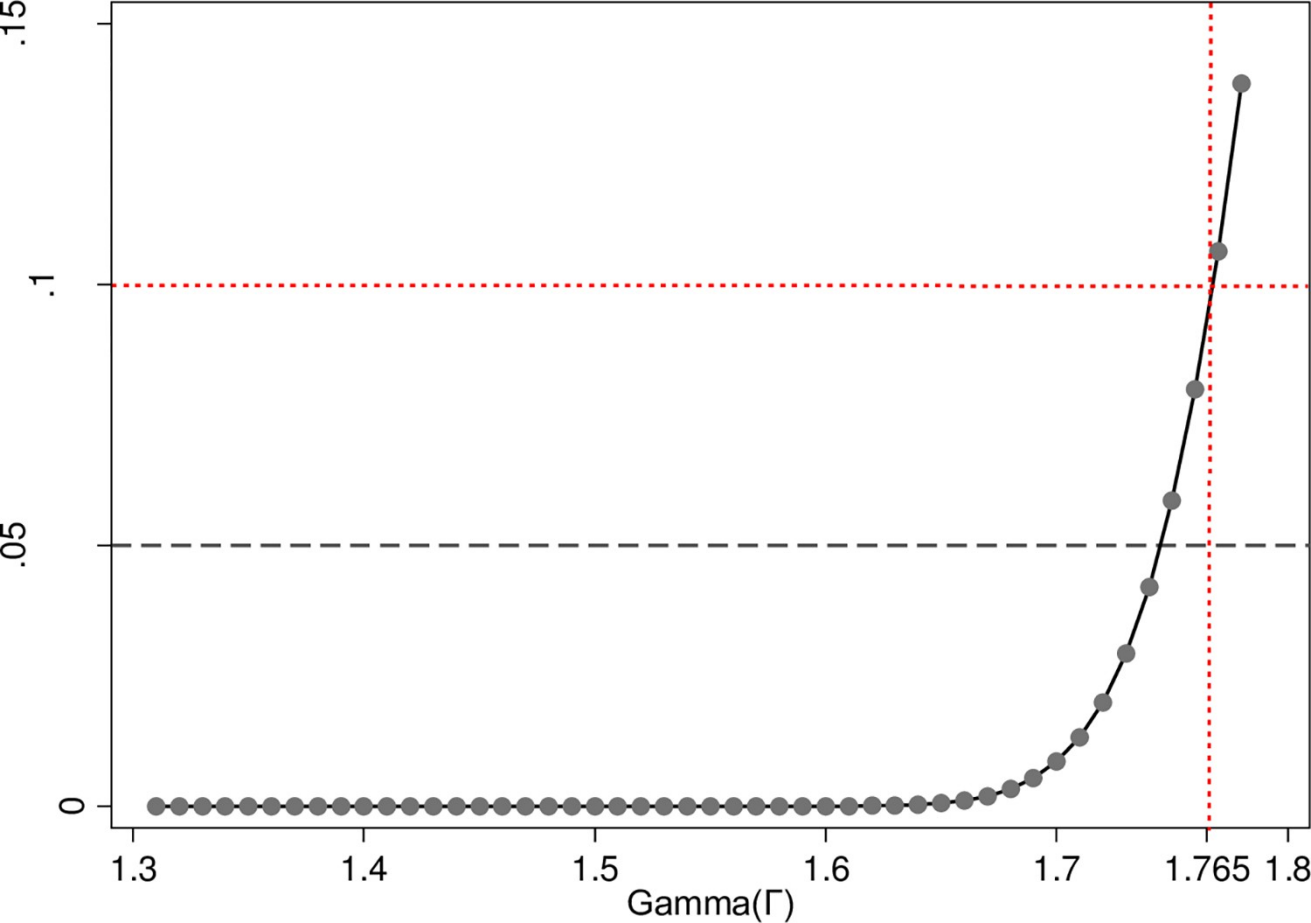
Rosenbaum sensitivity test for the matching model.

The issue of selection on unobservables [55] may challenge the results presented above. PSM is based on the key assumption of selection on observables or unconfoundedness [81], meaning that the treatment assignment is independent of the potential outcomes after controlling for observed covariates that affect both treatment status and the outcomes. Although I have carefully chosen and incorporated an array of pretreatment covariates and control variables to predict the propensity scores, unobservable confounding or omitted variables could still pose a significant challenge to the effectiveness of PSM procedures and the accuracy of estimations. This is because these confounders are not directly measured or included in the analysis, which can further introduce hidden bias and lead to incorrect inferences. In this regard, the Rosenbaum bound sensitivity test [82] is performed to determine the extent to which a hypothesized unobserved confounder might influence individuals’ choices of accessing the internet. Fig 5 indicates that the gamma coefficient corresponds to 1.765. According to Hu and Hibel [18], a rule of thumb for the social sciences is to set the range of gamma coefficient between 1 and 2; the closer this coefficient is to 2, the less likely an unovserable confounder exists. Therefore, the result suggests that the model used to predict propensity score can be considered as robust to “omitted-covariate bias” [18].

In addition to the sensitivity test, I also employ the widely used instrumental variable (IV) techniques for a robustness check. The IV method can address not only unobserved confounding or omitted variables, but also other sources of endogeneity [83] beyond the self-selection bias that PSM primarily focuses on and excels at handing, such as simultaneity or reverse causality. For example, it is reasonable to conceive that environmentalists who are knowledgeable about the environment may have a greater interest in seeking and consuming environmental information and participating in online discussions related to the environment [35], which may translate into more frequent and purposeful internet access and use. Hence, this study uses two instrumental variables to reduce the potential simultaneity as well as hidden bias: the number of internet broadband access port (see Model 3 in Table 5) and the number of internet users (see Model 4 in Table 5) at each province. The former indicates the availability and capacity of communication infrastructure in a certain region [34]; a higher value means people there could have more chances to access and use the internet frequently and conveniently [31]. The latter can be deemed as a strong peer influence that promotes internet access. These two IVs are not directly correlated with individuals’ levels of environmental knowledge. The results of the two-stage least square (2SLS) models are presented in Table 5, indicating that internet access significantly promotes respondents’ level of environmental knowledge after considering the simultaneity and omitted biases.

**Table 5:** Results of IV-2SLS models.

	Model 3	Model 4
Internet access	3.289** (1.132)	3.038* (1.224)
Pretreatment and control variables	YES	YES
Underidentification test: Kleibergen-Paap rk LM	38.557***	34.489***
Weak identification test: Kleibergen-Paap rk Wald F	38.667	34.870
Endogeneity test: Durbin-Wu-Hausman	3.388*	2.524*
*N*	9379	9379

Notes

Numbers in parentheses are robust standard errors.

*p < 0.05

**p < 0.001

***p < 0.001.

## Discussion

To reiterate, the main purpose of this study is to investigate the heterogeneous treatment effects of internet access and use on environmental knowledge in China. Overall, evidence on the internet access/use–environmental knowledge association exhibits positive and statistically significant direct effects of internet access. A more interesting finding is that this article reveals a negative distribution pattern of internet-based environmental knowledge returns; that is, individuals who are the most likely to benefit from the internet in terms of acquiring environmental knowledge are those who are the least likely to access it. Contrary to other studies on political, health, and science knowledge gaps [69,84–86], this result presents a much more positive outlook on the potential of the digital media to narrow the environmental knowledge gap [1,63].

To explain the findings, I use an additional variable to determine why digital media could narrow environmental knowledge gaps. I examine stratum-specific data on attention to environmental information. The results are presented (see S3 Fig), showing that individuals who rank in the higher propensity score strata (i.e., strata 11–15) are likely to pay more attention to environmental information through traditional media, while low- and middle-propensity members pay little attention to or do not care about the environmental information reported on traditional media. In this case, the high-propensity groups may have already acquired and accumulated environmental knowledge from traditional media; therefore, they may have encountered a ceiling effect [87–89] and thus may not able to increase their knowledge any further [90]. In contrast, as the internet coverage of environmental information increases, the positive effects of attention among low-propensity segments may become more pronounced. In this regard, the unlimited supply of content online coupled with the ease of retrieval of such information [91] provides an opportunity for disadvantaged groups to catch up with those in high-propensity strata.

In addition to this data-based explanation, it is plausible that the internet environment and online content drive the negative distribution of environmental knowledge. Specifically, the internet may resemble a relatively low-choice environment, where general users have very little control over the information to which they are exposed and, as such, learning occurs in a passive and incidental manner, i.e., learning without involvement or unanchored learning [92]. The magnitude of the outcomes of such passive learning, however, should vary between internet users. Notably, the colloquial nature of online expression [93] as well as the richness and edutainment narratives of environmental information [19] offer many opportunities for disadvantaged users to quickly reap knowledge benefits while surfing the internet for various purposes on a daily basis without spending much cognitive effort or even seeking out information out. However, advantaged users (i.e., who were more likely to access the internet in this study) might be less subject to passive learning partly because they have already acquired basic, nonsophisticated environmental knowledge and thus reached a ceiling, as mentioned previously; more importantly, they have a stronger capacity to opt into or out of information flows to fulfill their personal uses and gratifications [92,94]. Overall, internet-based environmental knowledge gaps are toward narrowing rather than widening.

A further interpretation of our result is that the observed negative distribution pattern is due to an unobserved selection mechanism through which individuals who are less likely to access the internet are more prone to overcome considerable odds to make up the “information deficit” [64] and, accordingly, are more rewarded by such unobserved/unexplained selectivity [16,17,95]. This selectivity, as speculated, might involve persons with low propensity to access and use the internet being aware of their “informational vulnerability” that forces them to voluntarily seek out information from multiple additional sources [66,96]; meanwhile, these alternative communication resources, for instance, interpersonal communication, create an environment in which disadvantaged groups may receive many more knowledge returns as a result of information repetition and the dissemination of new information [68].

Another primary goal of this study is to elucidate the internal mechanisms linking internet access/use to environmental knowledge. Drawing on negativity bias theory [44], this study proposes a conceptual framework that incorporates both the cognitive and perceptual factors. The study proves that the effect of internet access and use is indeed partially and substantially mediated by individual’s environmental awareness, perceived environmental threats, and satisfaction with governmental environmental protection. This multiple parallel mediation model [76] provides us with the opportunity to delve into the intricate pathways through which internet access/use influences environmental knowledge, as well as the interplay between online media content and audience reception [41].

However, it is important to acknowledge that the aforementioned mechanisms, to some extent, oversimplify and reduce the complexity of the relationship between media content and audience reception [24]. Media information is just one of several resources that contribute to citizens’ understanding of environmental issues [41]. In addition to providing easy access to a vast range of information and news on environmental topics, the internet serves as a communication infrastructure and information platform that hosts numerous specialized epistemic communities focused on specific environmental subjects; these communities bring together like-minded individuals, experts, and enthusiasts who contribute their experience and knowledge [97]. Moreover, the internet fosters a collaborative learning environment where participants from diverse backgrounds feel at ease seeking clarification, asking and answering questions, and engaging in informed discussions about the environment. Online social interactions play a pivotal role in knowledge-sharing communities [98], cultivating a participatory culture [99]. This culture not only builds bonding, shared solidarity, and a sense of reciprocity among community members [98,100], but also facilitates dialogue, encourages critical thinking, problem-solving, and the exchange of ideas, leading to a deeper understanding of environmental issues. Noteworthily, in recent years, there has been a growing scholarly interest in studying knowledge contribution behavior in various web-based knowledge-sharing platforms [97,98,100–103], aiming to identify predictors that explain users’ level of knowledge contribution and motivate their participation. Considering the importance of knowledge sharing in online collaborative learning, it is highly recommended that future studies explore knowledge-sharing communities specifically dedicated to environmental topics. Additionally, investigating the extent to which members’ knowledge contribution behavior contributes to their acquisition of environmental knowledge would be a valuable area of research.

In addition, this study reveals that the divide in internet access and use is still severe in China. Specifically, significant differences in internet access were found by gender, age, education, occupation, family income, residence type, marital status, and social contact but not innovativeness. This inform us the digital divide in internet access cannot be ignored and merits further investigation. More importantly, due to pretreatment heterogeneity, this study confirms that the OLS estimate of the effect of internet use on environmental knowledge is biased upward, which underscores the importance of adjusting for the population heterogeneity before we start to evaluate the treatment effects of internet access.

The limitations of this study should be noted. The present study is somewhat constrained by the use of secondary data. Although the CGSS 2013 includes a systematic and nationally representative sample, which increased the significance of the current research, it was not ideal for measuring some variables in the study. Particularly, the environmental knowledge measures were composed of a series of true-false questions, which fail to capture more abstract and in-depth aspects of knowledge [104,105]. Therefore, more nuanced measurement of environmental knowledge may be introduced in further studies. Moreover, while this study uses the propensity score approach, a series of techniques that have been claimed to be useful for causal inference under the counterfactual framework, it still faces some drawbacks. On one hand, due to the assumption of common support, excluding unplaced observations might lead to the sample selection bias, thereby undermining the generalizability and robustness of the results to a certain extent. On the other hand, while a series of robustness checks have been conducted to mitigate the issue of omitted variables and other endogeneity problems, these problems still exist and could bias the relationship of interest. It is suggested that future studies investigate the causal and net effect of internet use/access on environmental knowledge by employing more rigorous statistical techniques to rule out sources of endogeneity issues as much as possible.

## Supporting information

S1 Fig Indicators of environmental knowledge and descriptive statistics. (TIF)

S2 Fig Heterogeneous treatment of internet use/access on environmental knowledge (MS method). (TIF)

S3 Fig Proportion of different levels of attention by propensity score strata. (TIF)

S1 Table Pretreatment covariate means by propensity score strata and internet access (based on the SM method). (DOCX)
