# Supplementary figures and images for: Estimating heterogeneous effects of internet use on environmental knowledge: Taking population heterogeneity into consideration
Source: PLoS One. 2023 Jul 12;18(7):e0288495. doi: 10.1371/journal.pone.0288495 (PMC10337947; doi:10.1371/journal.pone.0288495)

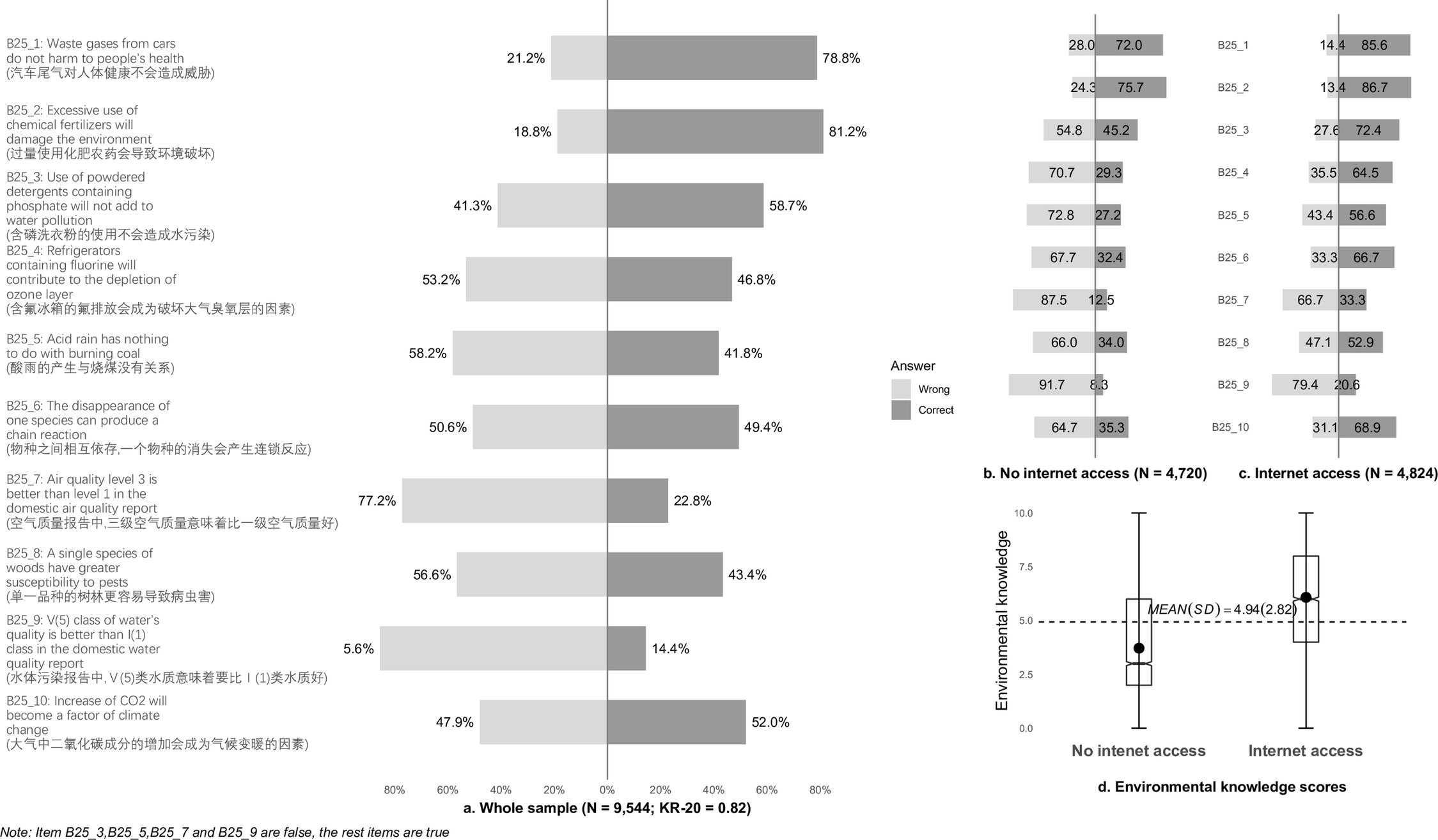

Supplement: S1 Fig — (TIF) [file pone.0288495.s001.tif]

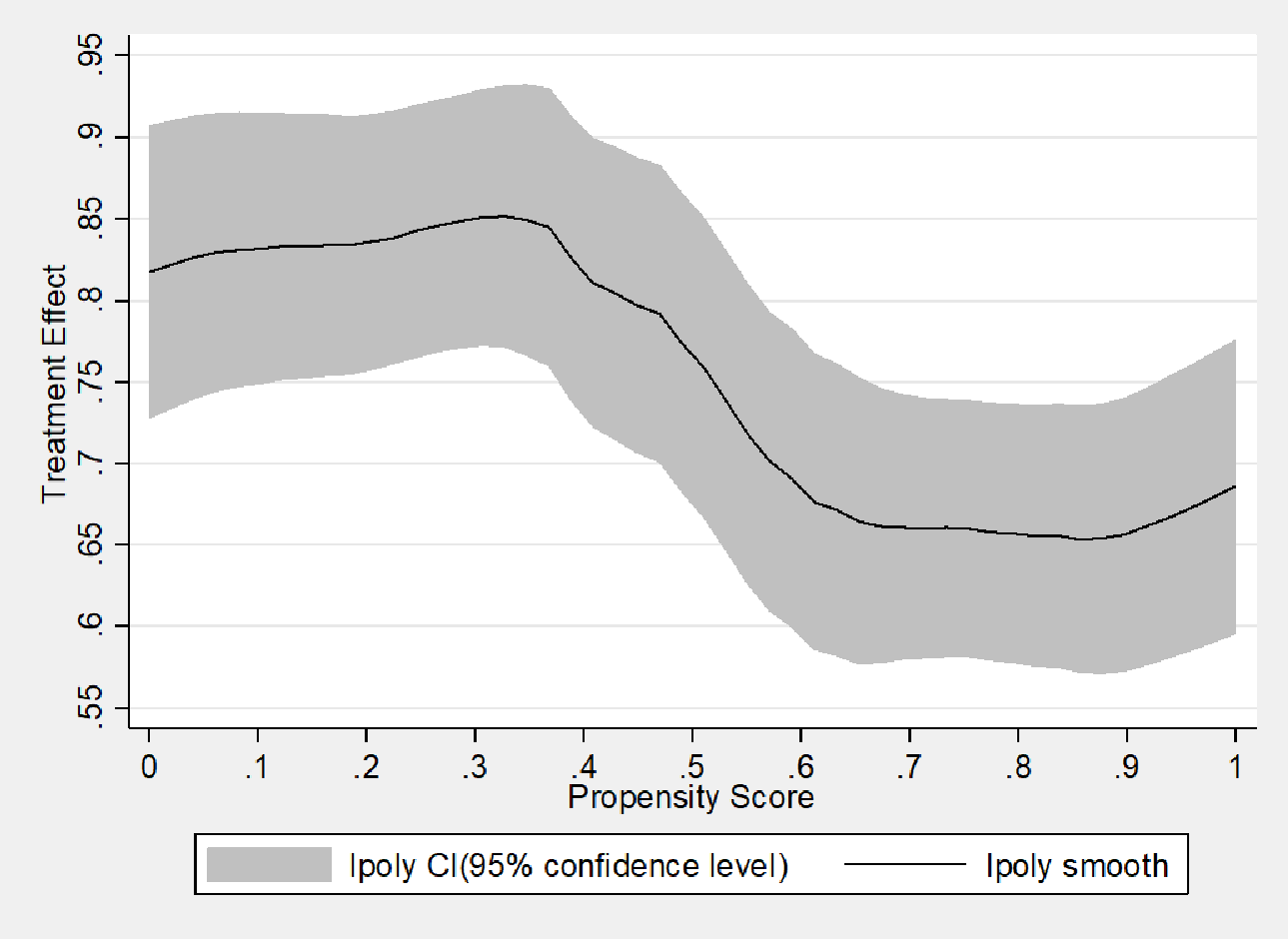

Supplement: S2 Fig — (TIF) [file pone.0288495.s002.tif]

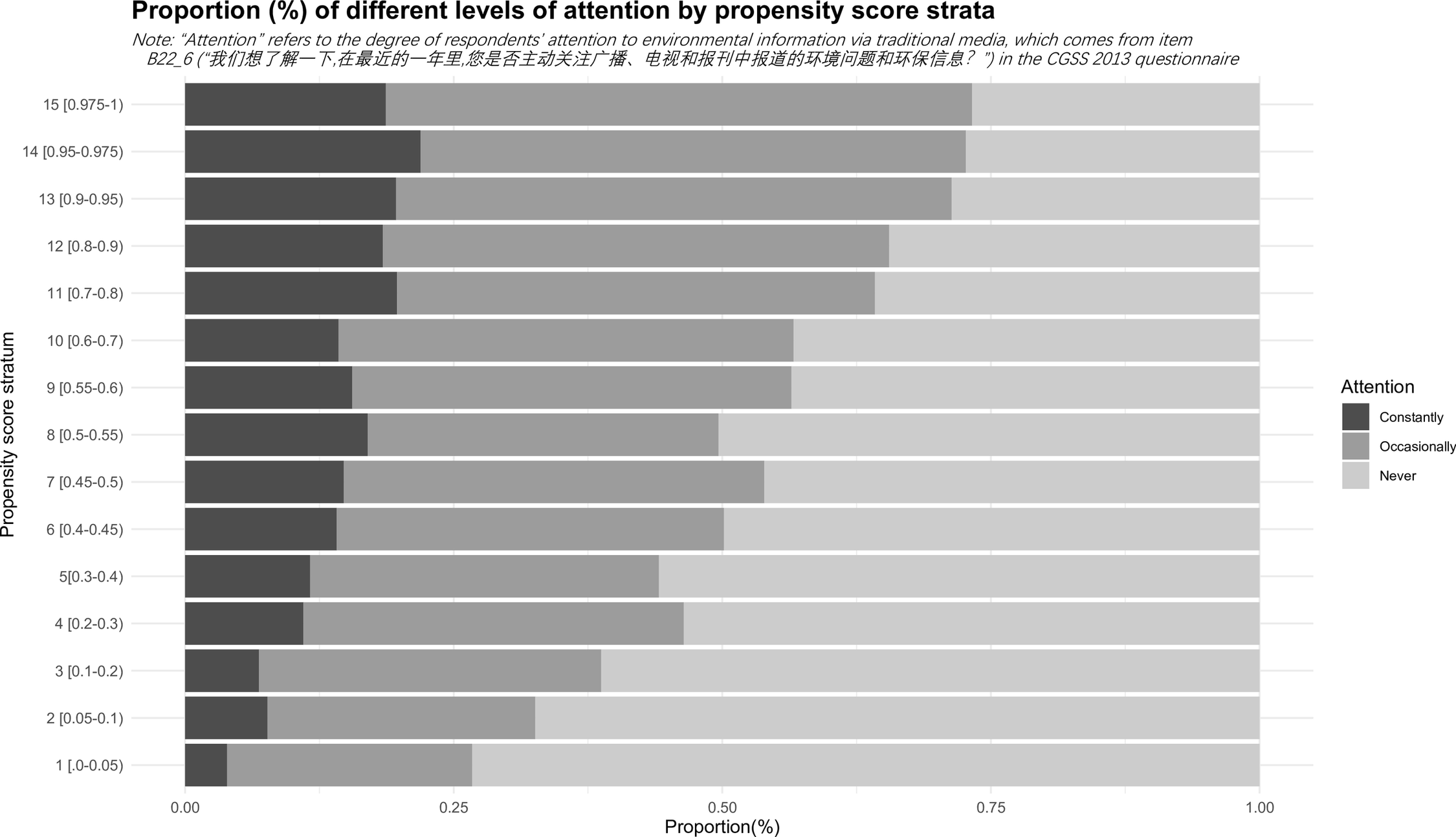

Supplement: S3 Fig — (TIF) [file pone.0288495.s003.tif]
