# Supplementary material for: Estimating heterogeneous effects of internet use on environmental knowledge: Taking population heterogeneity into consideration
Source: PLoS One. 2023 Jul 12;18(7):e0288495. doi: 10.1371/journal.pone.0288495 (PMC10337947; doi:10.1371/journal.pone.0288495)
Supplement: S1 Table — (DOCX) [file pone.0288495.s004.docx]

**S1 Table.** Pretreatment covariate means by propensity score strata and internet access (based on the SM method).

| Propensity score strata | | | | | | | | | | |
| --- | --- | --- | --- | --- | --- | --- | --- | --- | --- | --- |
|  | Strata 1 [.0-0.05) | | Strata 2 [0.05-0.1) | | Strata 3 [0.1-0.2) | | Strata 4 [0.2-0.3) | | Strata 5 [0.3-0.4) | |
| Variables | E(x)\|d=0 | E(x)\|d=1 | E(x)\|d=0 | E(x)\|d=1 | E(x)\|d=0 | E(x)\|d=1 | E(x)\|d=0 | E(x)\|d=1 | E(x)\|d=0 | E(x)\|d=1 |
| Female | 0.59 | 0.45 | 0.56 | 0.56 | 0.51 | 0.59 | 0.48 | 0.52 | 0.51 | 0.57 |
| Age | 4.84 | 4.81 | 4.35 | 4.54 | 3.99 | 4.09 | 3.83 | 4.09 | 3.51 | 3.57 |
| Educational year | 0.08 | 0.13 | 0.26 | 0.37 | 0.48 | 0.52 | 0.70 | 0.77 | 0.89 | 0.94 |
| Non-agricultural | 0.07 | 0.06 | 0.21 | 0.29 | 0.25 | 0.32 | 0.32 | 0.47 | 0.33 | 0.37 |
| Average annual household income (Ln) | 8.61 | 8.52 | 9.16 | 9.21 | 9.21 | 9.32 | 9.42 | 9.61 | 9.39 | 9.38 |
| Married | 0.99 | 0.96 | 0.98 | 1.00 | 0.99 | 0.98 | 0.99 | 0.99 | 0.97 | 0.98 |
| Social contact | -0.05 | 0.28 | -0.01 | 0.15 | 0.01 | -0.06 | 0.08 | 0.05 | -0.06 | 0.00 |
| Innovativeness | 4.11 | 3.77 | 4.11 | 4.17 | 3.97 | 4.04 | 4.13 | 4.05 | 4.05 | 3.91 |
| *N* | 1176 | 47 | 586 | 52 | 775 | 126 | 634 | 174 | 437 | 207 |
|  | Strata 6 [0.4-0.45) | | Strata 7 [0.45-0.5) | | Strata 8 [0.5-0.55) | | Strata 9 [0.55-0.6) | | Strata 10 [0.6-0.7) | |
| Variables | E(x)\|d=0 | E(x)\|d=1 | E(x)\|d=0 | E(x)\|d=1 | E(x)\|d=0 | E(x)\|d=1 | E(x)\|d=0 | E(x)\|d=1 | E(x)\|d=0 | E(x)\|d=1 |
| Female | 0.46 | 0.40 | 0.49 | 0.45 | 0.45 | 0.49 | 0.48 | 0.50 | 0.54 | 0.52 |
| Age | 3.64 | 3.52 | 3.51 | 3.47 | 3.43 | 3.27 | 3.22 | 3.25 | 3.31 | 3.07 |
| Educational year | 1.16 | 1.08 | 1.15 | 1.10 | 1.24 | 1.15 | 1.28 | 1.36 | 1.59 | 1.46 |
| Non-agricultural | 0.44 | 0.40 | 0.43 | 0.41 | 0.50 | 0.38 | 0.46 | 0.47 | 0.61 | 0.53 |
| Average annual household income (Ln) | 9.55 | 9.49 | 9.68 | 9.70 | 9.68 | 9.71 | 9.63 | 9.57 | 9.73 | 9.67 |
| Married | 0.97 | 0.96 | 0.95 | 0.97 | 0.95 | 0.98 | 0.97 | 0.97 | 0.96 | 0.97 |
| Social contact | 0.06 | 0.02 | 0.03 | 0.14 | 0.01 | 0.14 | 0.21 | 0.18 | -0.03 | -0.07 |
| Innovativeness | 3.91 | 4.09 | 3.98 | 3.91 | 4.00 | 3.85 | 3.98 | 3.98 | 3.96 | 4.11 |
| *N* | 205 | 114 | 190 | 155 | 152 | 136 | 97 | 128 | 208 | 394 |
|  | Strata 11 [0.7-0.8) | | Strata 12 [0.8-0.9) | | Strata 13 [0.9-0.95) | | Strata 14 [0.95-0.975) | | Strata15 [0.975-1) | |
| Variables | E(x)\|d=0 | E(x)\|d=1 | E(x)\|d=0 | E(x)\|d=1 | E(x)\|d=0 | E(x)\|d=1 | E(x)\|d=0 | E(x)\|d=1 | E(x)\|d=0 | E(x)\|d=1 |
| Female | 0.47 | 0.44 | 0.51 | 0.46 | 0.56 | 0.48 | 0.33 | 0.43 | 0.29 | 0.45 |
| Age | 3.08 | 2.94 | 2.62 | 2.73 | 2.56 | 2.30 | 2.75 | 2.31 | 1.29 | 1.55 |
| Educational year | 1.68 | 1.60 | 1.84 | 1.94 | 2.26 | 2.12 | 2.83 | 2.46 | 2.14 | 2.76 |
| Non-agricultural | 0.56 | 0.51 | 0.63 | 0.64 | 0.76 | 0.58 | 0.83 | 0.74 | 0.71 | 0.77 |
| Average annual household income (Ln) | 9.89 | 9.90 | 9.77 | 9.89 | 9.95 | 9.92 | 10.02 | 10.05 | 10.18 | 10.31 |
| Married | 0.96 | 0.96 | 0.95 | 0.91 | 0.88 | 0.81 | 0.75 | 0.76 | 0.29 | 0.39 |
| Social contact | 0.20 | 0.14 | 0.32 | 0.02 | 0.22 | 0.05 | 0.03 | 0.06 | -0.26 | -0.03 |
| Innovativeness | 3.67 | 3.86 | 3.66 | 3.86 | 4.03 | 3.88 | 4.25 | 3.91 | 3.86 | 4.04 |
| *N* | 131 | 467 | 76 | 603 | 34 | 430 | 12 | 449 | 7 | 1342 |

*Notes*: Numbers in each cell denote mean pretreatment covariate values. Values for all the occupation types are not shown for the sake of parsimonious presentation. E(x)|d=0 indicates the mean of X for respondents who are not access to the internet, and E(x)|d=1 indicates the mean of X for respondents who had and having been accessed to the internet. Two-sample T tests are performed to make sure all of the pretreatment covariates are balanced with each propensity score stratum.
